# Functional and molecular characterization of inherited platelet disorders in the Iberian Peninsula: results from a collaborative study

**DOI:** 10.1186/s13023-014-0213-6

**Published:** 2014-12-24

**Authors:** Isabel Sánchez-Guiu, Ana I Antón, José Padilla, Francisco Velasco, José F Lucia, Miguel Lozano, Ana Rosa Cid, Teresa Sevivas, María F Lopez-Fernandez, Vicente Vicente, Consuelo González-Manchón, José Rivera, María L Lozano

**Affiliations:** Servicio de Hematología y Oncología Médica, Hospital Universitario Morales Meseguer, Centro Regional de Hemodonación, Universidad de Murcia, IMIB-Arrixaca, Murcia, 30003 Spain; Servicio de Hematología y Hemoterapia, Instituto Maimonides de Investigación Biomédica de Córdoba (IMIBIC), Hospital Universitario, Córdoba, Spain; Servicio Hematología y Hemoterapia, Hospital Universitario Miguel Servet, Zaragoza, Spain; Servicio de Hemoterapia y Hemostasia, Hospital Clínico, Barcelona, Spain; Unidad de Hemostasia y Trombosis, Servicio Hematología y Hemoterapia, Hospital Universitario Politécnico de la Fe, Valencia, Spain; Serviço de Hematologia do Centro Hospitalar e Universitário de Coimbra, Coimbra, Portugal; Servicio Hematología y Hemoterapia, Complejo Hospitalario Universitario A Coruña, La Coruña, Spain; Departament Cellular and Molecular Medicine, Centro de Investigaciones Biológicas (C.S.I.C.),CIBER de Enfermedades Raras, Madrid, Spain

**Keywords:** Bernard Soulier syndrome, Glanzmann thrombasthenia, Inherited platelet disorders, Chediak-Higashi syndrome, Hermansky Pudlak syndrome, ANKRD26, Congenital amegakaryocytic thrombocytopenia

## Abstract

**Background:**

The diagnostic evaluation of inherited platelet disorders (IPDs) is complicated and time-consuming, resulting in a relevant number of undiagnosed and incorrectly classified patients. In order to evaluate the spectrum of IPDs in individuals with clinical suspicion of these disorders, and to provide a diagnostic tool to centers not having access to specific platelets studies, we established the project “Functional and Molecular Characterization of Patients with Inherited Platelet Disorders” under the scientific sponsorship of the Spanish Society of Thrombosis and Haemostasis.

**Patients/methods:**

Subjects were patients from a prospective cohort of individuals referred for clinical suspicion of IPDs as well as healthy controls. Functional studies included light transmission aggregation, flow cytometry, and when indicated, Western-blot analysis of platelet glycoproteins, and clot retraction analysis. Genetic analysis was mainly performed by sequencing of coding regions and proximal regulatory regions of the genes of interest.

**Results:**

Of the 70 cases referred for study, we functionally and molecularly characterized 12 patients with Glanzmann Thrombasthenia, 8 patients with Bernard Soulier syndrome, and 8 with other forms of IPDs. Twelve novel mutations were identified among these patients. The systematic study of patients revealed that almost one-third of patients had been previously misdiagnosed.

**Conclusions:**

Our study provides a global picture of the current limitations and access to the diagnosis of IPDs, identifies and confirms new genetic variants that cause these disorders, and emphasizes the need of creating reference centers that can help health care providers in the recognition of these defects.

## Background

Inherited platelet disorders (IPDs) constitute a group of relatively uncommon isolated diseases usually characterized by a lifelong mild to moderate bleeding diathesis, with a true incidence still unknown. Although individual IPDs are rare and a population based study on the overall prevalence of these disorders has never been undertaken, in aggregate they may be as prevalent as von Willebrand disease (vWD), the most common inherited bleeding disorder [1-3].

A few classic IPDs such as Bernard–Soulier Syndrome (BSS) and Glanzmann Thrombasthenia (GT) have been molecularly characterized for over 20 years, while others such as inherited disorders of signaling pathways downstream from membrane receptors are still in many cases difficult to diagnose since the molecular and genetic mechanisms are in the vast majority of patients unknown [4]. Following the suspicion of an IPD, the diagnosis should be attained by a combination of an appropriate personal and family history, a physical examination, standard laboratory studies, including an assortment of platelet morphologic and functional studies, and ideally, confirmatory, targeted-gene analyses [5-8]. However, the identification of many platelet disorders has been hampered by the lack of common international consensus criteria for diagnostic algorithms, and also by the limited access to specific platelets studies and to molecular tests which are usually performed only by a relatively small number of highly specialized laboratories [8]. Additionally, it’s been long established that platelet functional studies are to be performed within a limited time after blood collection, although the feasibility of performing those tests at a more extended time lapse when evaluating severe platelet defects has rarely been addressed [9]. Consequently, diagnosed cases are frequently clustered around such centers, while elsewhere >50% of patients who are likely to be affected remains partially characterized and without a molecular diagnosis [10,11]. As information develops from the study of IPDs, more patients will be identified, expanding our understanding about normal platelet biochemistry and physiology, and our knowledge of the functional network and critical domains of specific platelet elements. These investigations will provide patients with clearer clinical diagnoses, and will permit to identify affected family members, as well as benefit patients from genetic counseling. Moreover, these studies are essential to distinguish these diseases from acquired platelet disorders to avoid unnecessary and potentially harmful treatments, and to educate patients on when to seek for specific therapies and about the effect of lifestyle on hemostasis [12,13]. Although sporadic IPDs from the Iberian Peninsula have been described, until recently, no reference laboratory to assist physicians on the diagnosis of patients with suspected IPDs was available. Therefore, to respond to such a challenge, and with the aims to (1) evaluate the accuracy of previous diagnoses of IPD by non-specialized diagnostic laboratories, (2) analyze the feasibility of performing reliable platelet functional studies within the 24 hours from phlebotomy, under specific conditions, (3) and to confirm a diagnosis of IPD at a molecular level we undertook this study. Presented information includes data from the “Functional and Molecular Characterization of Patients with Inherited Platelet Disorders” a collaborative project that was undertaken under the scientific sponsorship of the Spanish Society of Thrombosis and Hemostasis. The main part of this manuscript focuses on describing our findings from patients diagnosed with two severe autosomal recessive IPDs affecting the platelet adhesion and aggregation, BSS and GT respectively [14,15].

## Methods

### Patient selection and blood drawing

In the course of the last five years, samples from 70 unrelated patients with a clinical suspicion of IPD from different Spanish and Portuguese hospitals have been analyzed for this study. Venous blood was drawn from each patient and a parallel healthy control that had not taken antiplatelet medication for at least 2 weeks, and samples from both individuals were delivered within 18–24 hours by express courier at room temperature to Murcia, Spain, where all further studies were done. Moreover, fresh blood was taken from a healthy volunteer from our facility each day that functional studies were performed, and served as an additional control. Seventeen patients had their blood drawn at our institution, and in those cases, blood specimens were only collected from fresh (not parallel) controls. Blood samples were collected into commercial 7.5% K3 EDTA tubes (for complete blood count [CBC], and DNA & RNA isolation), and into buffered 3.2% sodium citrate (for platelet functional studies) using a 20-gauge needle. Immediately after blood collection, all tubes were mixed by gentle inversion at least 6 times (and discarded if there was any evidence of clotting), and samples were maintained at room temperature (20-25°C) during shipping.

All individuals gave informed consent; the sampling from the children was carried out with the permission of the parents. Controls, patients, and relatives were fully informed of the aim of this study, which was performed according to the declaration of Helsinki, as amended in Edinburgh in 2000. This study obtained approval from the Reina Sofia Hospital Ethics Committee.

The bleeding tendency of patients and their relatives was recorded by the medical doctors using a bleeding score (BS) questionnaire that recorded the most frequent and typical symptoms from 0 to 3 [16,17]. The recorded data were then processed providing a numerical assessment of bleeding severity (Table 1). Considering that sending samples to a distant laboratory might pose the problem of missing mild platelet defects, we restricted the study to patients with significant bleeding history. In the absence of specific features (see below) we required for a patient to be considered eligible to be included in this study, (1) a bleeding score score ≥3 in male subjects, ≥5 in female subjects, and ≥2 in the pediatric population, and (2) more than 3 bleeding symptoms in the adult population. Exceptions to these inclusion criteria were the 7 patients presenting specific features characteristic of IPDs: 2 patients with hypopigmentation and giant granules in polymorphonuclear leukocytes characteristic of Chediak-Higashi syndrome (CHS), 1 patient with oculocutaneous albinism and nystagmus suggestive of Hermansky-Pudlak syndrome (HPS), 2 patients with macrothrombocytopenia and leukocyte inclusion bodies typical of MYH9-related disorder (MYH9-RD), 1 patient with a hypocellular bone marrow exhibiting a near complete absence of megakaryocytes distinctive of congenital amegakaryocytic thrombocytopenia (CAMT), and 1 patient with a three-generation family history of autosomal dominant thrombocytopenia with normal mean platelet volume (MPV), suggestive of defects in megakaryocyte maturation.

**Table 1:** **Clinical characteristics and bleeding score of patients included in the study**

	**GT**	**BSS**	**CHS**	**HPS**	**MYH9-RD**	**CAMT**	**THC2**	**SSD**	**Presumably misdiagnosed**	**Total**
	**n = 13**	**n = 8**	**n = 2**	**n = 1**	**n = 2**	**n = 1**	**n = 1**	**n = 19**	**n = 23**	**n = 70**
Sex (F/M)	9/4	6/2	1/1	0/1	1/1	0/1	1/0	13/6	16/7	47/23
Age at clinical suspicion/diagnosis (median ± SD)	10 ± 10	12 ± 5	5 ± 1	2	30 ± 21	6	12	29 ± 24	13 ± 19	12 ± 19
Age at referral for study (median ± SD)	32 ± 18	40 ± 7	12 ± 11	28	34 ± 16	21	36	38 ± 21	36 ± 19	35 ± 18
Mean BS	10.5	11.8	0	6	0.5	8	6	8.6	6.1	7.9
% Patients with BS ≥ 2										
• Epistaxis	54	100	0	0	0	100	0	21	30	38
• Cutaneous symptoms	58	50	0	0	0	0	0	47	25	38
• Minor wounds	8	13	0	0	0	0	0	26	16	15
• Oral cavity bleeding	46	38	0	0	0	0	0	21	10	22
• Gastrointestinal bleeding	31	33	0	0	0	0	0	26	21	23
• Tooth extraction	33	57	0	100	0	N/A	N/A	46	31	38
• Surgery	40	71	0	100	0	100	N/A	73	38	51
• Menorrhagia	100	100	0	N/A	0	N/A	100	67	62	76
• Postpartum hemorrhage	33	75	0	N/A	0	N/A	0	40	33	39
• Hematomas or hemarthrosis	8	0	0	0	0	0	0	11	16	10

The Table reflects the clinical characteristics of patients that were confirmed to have a final diagnosis consistent or not (in such case considered “presumably misdiagnosed”) with the previously suspected IPD, the total bleeding score, and the percentage of patients with clinical significant bleeding (grades 2 or 3) for each symptom. For each symptom the grades of bleeding severity ranged from 0 (absence of symptoms) to 3. Grade 1 was given when a patient reported the presence of bleeding, grade 2 if the symptom required evaluation by a physician but no active intervention, and grade 3 if there was some type of intervention. The final bleeding score was generated by summing the severity of all bleeding symptoms reported by the patient [16,17].

*Abbreviations*: *BS* Bleeding score, *BSS* Bernard Soulier syndrome, *CAMT* congenital amegakaryocytic thrombocytopenia, *CHS* Chediak-Higashi syndrome, *F* Female, *GT* Glanzmann Thrombasthenia, *HPS* Hermansky-Pudlak syndrome, *M* Male, *MYH9-RD* MYH9 related disorder, *N/A* not applicable, *SSD* platelet secretion and signal transduction defects, *THC2* thrombocytopenia 2.

Before any additional testing, it was mandatory to have results from the following tests: basic biochemical tests including renal and liver function, prothrombin time, activated partial thromboplastin time, CBC, MPV, and morphological examination of a blood smear. In patients not exhibiting thrombocytopenia, additionally Clauss fibrinogen, factor VIII: C activity, von Willebrand factor (vWF) antigen, and ristocetin cofactor activity assay were studied. Patients were excluded from further laboratory testing after a diagnosis of acquired defects or vWD. Subject diagnoses were based on the opinion of the referring physician, and the BS questionnaire, and medical records were examined by two physicians from our facility who also reviewed laboratory data. Definitive diagnoses of IPDs were compared to the clinical suspicion by the referring physician, and sub-classified as previously being accurately diagnosed or as presumably misdiagnosed, based on platelet functional studies as well as on molecular findings. Initial evaluation at our institution also included platelet aggregation, glycoprotein expression, and analysis of activation markers by flow cytometry, and if indicated, specialized tests were performed, including clot retraction, and molecular studies.

### Platelet functional studies

Platelet rich plasma (PRP) was prepared by centrifugation of citrated whole blood at 150 × g for 10 min and the top two-thirds removed by careful pipetting into a separate tube. Platelet poor plasma (PPP) was prepared by a further centrifugation step of the same tube at 1000 × g for 20 min. PRP from citrated blood samples that appeared macrothrombocytopenic on blood smears were prepared by means of tilting the tube to an angle of 45° for 1-2 h [18].

Light transmission aggregometry (LTA) was performed as described [19] using undiluted PRP when the platelet concentration of patient’s and control’s PRP samples were in the range of 200 and 600 × 10^9^ L^−1^ [20]. When the patient’s PRP count was below 150 × 10^9^ L^−1^, we diluted the control’s sample with autologous PPP, in order to have similar optical densities (±10%). Time course changes in the maximal percentage of light transmission of PRP over baseline (PPP) were recorded for 300s using an Aggrecorder II aggregometer (Menarini Diagnostics, Florence, Italy). Agonists used for LTA included: 2 μg mL^−1^ collagen, 10 μM ADP; 1.6 mM arachidonic acid, 1.25 mg mL^−1^ ristocetin, and 25 μM Thrombin Receptor Activating Peptide (TRAP). In patients with clinical suspicion of GT, responses to 100 nM phorbol myristate acetate (PMA) were also analyzed; in those with suspicion of BSS, only aggregatory responses to ristocetin and TRAP were assessed. Some subjects were not tested with all agonists. LTA samples were preincubated (37°C, 3 min), and all tracings were inspected by the same person (JR), who provided an overall interpretative comment. Platelet aggregation was considered abnormal if it was reversible, or if the maximum amplitude was <40% of the paired parallel control drawn the same day and shipped under similar conditions than samples from each patient. If the samples from such paired control from the referring center exhibited considerable weaker responses to 25 μM TRAP than those of the fresh control (<30%), the tubes were discarded for functional analysis, and further samples were requested for analysis.

For flow cytometry, citrated whole blood diluted 1:10 in saline buffer was assessed for expression of major platelet membrane glycoproteins (GP) that support platelet adhesion and aggregation (GPIa, GPIbα, α_IIb_, β_3_) through a direct standard flow cytometry technique with appropriate labelled monoclonal antibodies in a FACScalibur platform (Becton Dickinson, San Jose, CA). PRP was used to study platelet activation markers (P-selectin, granulophysin [stimulated with 25 μM TRAP]), and activated α_IIb_β_3_ complex (unstimulated and activated with 100 nM PMA or 10 μM ADP). Monoclonal antibodies (Becton Dickinson) used were FITC*CD49b (GPIa), FITC*CD42b (GPIbα), PE*CD41a (α_IIb_), FITC*CD61 (β_3_), FITC*PAC-1 (activation-specific antiα_IIb_β_3_ antibody), PE*CD62 (P-selectin), and PE*CD63 (granulophysin). For each sample run, data acquisition of 10,000 events was gated on forward and side-angle light scatter with gains adjusted to include the platelet population. Then, the fluorescence of stained platelets was analysed (CellQuest software, Becton Dickinson) to obtain both the percentage of positively stained cells and the mean fluorescence intensity (MFI).

For patients with the clinical suspicion of GT, we additionally tested the *in vitro* haemostatic response through the “clot retraction assay”. PRP samples were diluted in homologous PPP to final platelet counts of 300 × 10^9^L^−1^ and incubated in glass tubes for 5 minutes at 37°C. Clot formation was induced by calcium chloride (8 mM L^−1^ final concentration), and samples were photographed and weighed after 1 hour of incubation at 37°C [21].

Samples from patients showing reduced/absent α_IIb_β_3,_ or GPIb/IX expression underwent Western blot analysis of platelet glycoproteins, by standard procedures using specific antibodies recognizing α_IIb_ (132.1, Blood Research Institute, Milwaukee, WI), β_3_ (AP3, Blood Research Institute, Milwaukee, WI), and GP Ibα (LJ-Ib10, La Jolla, CA).

### Molecular characterization

DNA and RNA were isolated from each patient by standard procedures, and stored frozen until use. Molecular analysis of candidate genes was done in patients with a laboratory diagnosis of GT or BSS (see below), and also in patients with clinical and laboratory suspicion of MYH9-RD, CHS, HPS, Platelet von Willebrand disease (PvWD), CAMT, and thrombocytopenia 2 (THC2).

To detect mutations in *ITGA2B* (ENSG00000005961) or *ITGB3* (ENSG00000259207), total RNA from peripheral blood was used for cDNA synthesis using the Superscript First Strand Synthesis System for RT-PCR (Invitrogen, Paisley, UK), following the manufacturer’s protocol. Using specific oligonucleotides and conditions (primer sequences and conditions are available upon request) we amplified and sequenced *ITGA2B* and *ITGB3* by Sanger’s approach. Every mutation found in cDNA was confirmed on DNA samples.

The *GP1BA* (ENSG00000185245), *GP1BB* (ENSG00000203618) and *GP9* (ENSG00000169704) genes were screened for mutations using genomic DNA. Genomic fragments were analysed by polymerase chain reaction (PCR) using primers designed specifically to amplify the coding regions (primer sequences and conditions are also available upon request). PCR products were sequenced in the ABI 3130xl genetic analyser (Applied Biosystems, Carlsbad, CA).

Patients with the clinical suspicion of other IPDs were studied and screened for mutations in the specific candidate gene. Thus, all exons and intron-boundaries of *HPS1* (ENSG00000107521) were sequenced by Sanger’s method in two patients with the clinical suspicion of HPS; in one of these patients *HPS4* (ENSG00000100099) was additionally sequenced. RNA from patients with the suspicion of CHS and MYH9-RD was extracted, and cDNA was amplified and sequenced seeking mutations in *LYST/CHS1* (ENSG00000143669) and *MYH9* (ENSG00000100345)*,* respectively. When the clinical suspicion was PvWD, CAMT, or THC2, the molecular approach contemplated the direct analysis of the most affected DNA regions in these disorders: exon 2 of *GP1BA* in PvWD [22], the first 3 exons of *MPL* (ENSG00000117400) gene in CAMT [23], and the 5′UTR region of *ANKRD26* (ENSG00000107890) in THC2 [24]. In one patient with clinical suspicion of BSS not confirmed by functional and molecular studies, exome sequencing was performed (Agilent SureSelect Capture v4 + UTRs and Genome Analyzer IIx platform, Agilent Technologies, Santa Clara, CA). Using RUbioSeq software [25] sequencing data were aligned to the human reference genome (GRCh37) and exome variant analysis and functional prediction were performed with the default parameters for somatic variation analysis.

## Results

Of the 70 patients enrolled in this study, molecular characterization of GT was achieved in 12 patients, while the causative mutation/s was recognized in 8 cases of BSS, in 2 with CHS, 1 with HPS, 3 with MYH9-RD, 1 with CAMT, and 1 with THC2 (10 men and 18 women) (Table 2). In these patients, the median age at first suspicion of IPD was 10 years; the median age at which an IPD was molecularly confirmed was 34 years. Overall, 12 novel mutations have been identified in this study. In about two-third of the cases that had been referred with a clinical suspicion/diagnosis of IPD, our functional and molecular approach confirmed a definitive diagnosis of platelet disorder. However, the median age at which IPD was confirmed in these 47 patients was 35 years, indicating that there was a significant delay (24 years) from the time an IPD was first suspected to the time confirmatory evaluation was requested. In about one-third of the cases (n = 27) that had been suspected of having IPD during a median period of 23 years (the median age when IPD was suspected and when specific laboratory evaluation was performed was 13 and 36 years, respectively) our approach could not confirm such diagnosis, and were therefore categorized as presumably misdiagnosed. Table 2 reflects the observed misdiagnosis rate according to specific platelet disorders that had been clinically suspected.

**Table 2:** **Molecular and functional characterization of patients with IPDs**

**Diagnostic suspicion at referral**	**n**	**Confirmed IPDs (n = 47)**		**Presumably misdiagnosed (n = 23)**	
		**Phenotype/Functional**	**Molecular diagnosis**	**Cases**	**Presumptive diagnosis**
**Signaling and/or secretion disorder**	25	19 (76%)	0	6 (24%)	No platelet defect
**Glanzmann thrombasthenia**	20	13 (65%)	12	7 (35%)	Signaling and/or secretion disorders
**Bernard-Soulier syndrome**	13	8 (61.5%)	8	5 (38.5%)	MUO (n = 4)
					MYH9-RD (n = 1)*
**Chediak-Higashi syndrome**	2	2 (100%)	2	0	-
**Hemansky-Pudlak syndrome**	2	1 (50%)	1	1 (50%)	No molecular confirmation of disease
**MYH9-RD**	4	2 (50%)	3*	2 (50%)	MUO
**Platelet von Willebrand disease**	2	0	-	2 (100%)	No definitive diagnosis
**Congenital Amegakaryocytic Thrombocytopenia**	1	1	1	0	-
**Thrombocytopenia 2**	1	1	1	0	-
**All IPDs Referred**	**70**	**47 (67.1%)**	**28 (40.0%)**	**23 (32.9%)**	

*Abbreviation*: *IPDs* inherited platelet disorders, *MYH9-RD* MYH9 related disorder, *MUO* macrothrombocytopenia of unknown origin.

*One of the patients referred with a clinical suspicion of BSS was diagnosed as having MYH9-related disorder.

### *Glanzmann thrombasthenia*

Thirteen patients were diagnosed with GT (4 men and 9 women, with a median age of 32 years) (Tables 1 and 3). The disorder was readily identifiable in the presence of a normal platelet number (109-280 × 10^9^ platelet L^−1^) and size, and by platelet function testing, consisting in severely impaired aggregation in response to all physiological agonists, and also to PMA (Table 4). Ristocetin-induced platelet agglutination (RIPA) was normal or reduced to some extent (74 ± 25%), and GPIbα and GPIX expression was normal in all patients, while clot retraction was consistently abnormal. On flow cytometry 8 patients (GT-2, GT-4, GT-5, GT-8, GT-9, GT-10, GT-12 and GT-13) were diagnosed as type I GT (<5% of α_IIb_β_3_ expression), 4 (GT-1, GT-3, GT-6 and GT-7) as type II GT (α_IIb_β_3_ expression between 5% and 20%), while 1 patient (GT-11) was classified as variant GT (50% expression but receptor being not functional according to LTA and clot retraction). All patients exhibited no binding of PAC-1 activation-specific anti- α_IIb_β_3_ antibody upon stimulation with ADP or TRAP. In platelet lysates from type II patients, faint but detectable α_IIb_β_3_ bands of the expected apparent molecular weight were seen, while platelets from the patient with variant GT showed reduced (50% of normal) levels of these proteins (data not shown).

**Table 3:** **Molecular diagnosis of patients with Glanzmann Thrombasthenia**

**Patients with molecular diagnosis**		**Gene**	**Inheritance**	**Mutation**	**Reference**
**GT-1**	GT II	*ITGB3*	Compound Heterozygous	p.Met124Val	[30]
				c.774-775delTG	new
**GT-2**	GT I	*ITGA2B*	Compound Heterozygous	p.Leu183Pro	[26]
				c.2473_2481delinsTCACCTGGTC	new
**GT-3**	GT II	*ITGA2B*	Homozygous	p.Cys674Arg	[27]
**GT-4**	GT I	*ITGB3*	Homozygous	p.Tyr190Cys	new
**GT-5**	GT I	*ITGA2B*	Compound Heterozygous	p.Glu507stop	new
				c. 2637delC	new
**GT-6**	GT II	*ITGA2B*	Compound Heterozygous	p.Val951Met	[31]
				p.Ala958Thr	[31]
				p.Glu507stop	new
**GT-7**	GT II	*ITGB3*	Homozygous	p.Met118Arg	[32]
**GT-8**	GT I	*ITGA2B*	Homozygous	c.2965delG	new
**GT-9**	GT I	*ITGA2B*	Homozygous	c.692insT	[28]
**GT-10**	GT I	*ITGA2B*	Homozygous	c.1599delAT	new
**GT-11**	GT Variant	*ITGB3*	Compound Heterozygous	p.Tyr190Cys	new
				p.Leu196Pro	[29]
**GT-12**	GT I	*ITGB3*	No mutation found	-	-
**GT-13**	GT I	*ITGA2B*	Homozygous	p.Trp51Stop	new

*Abbreviation*: *BS* bleeding score, *GT* Glanzmann Thrombasthenia.

**Table 4:** **Maximal aggregation (%) of patients and controls**

	**Platelet count (×10** ^**9**^ ** L** ^**−1**^ **)**	**TRAP (25 μM)**	**ADP (10 μM)**	**Ristocetin (1.25 mg mL** ^**−1**^ **)**	**Arachidonic acid (1.6 mM)**	**Collagen (2 μg mL** ^**−1**^ **)**	**PMA (100 nM)**
**GT (n = 13)**	171.7 ± 42.5	18.3 ± 4.5	3.0 ± 5.2	74.1 ± 24.7	9.8 ± 5.0	8.2 ± 9.6	8.8 ± 4.4
**BSS (n = 8)**	*	49.4 ± 21.1	-	4.4 ± 2.5	-	-	-
**SSD (n = 19)**	197.6 ± 77.8	80.7 ± 11.2	53.9 ± 27.0	84.6 ± 17.8	72.1 ± 25.8	45.7 ± 36.4	-
**Parallel controls (n = 31)‡**	216.3 ± 48.9	83.7 ± 12.7	78.1 ± 16.8	80.3 ± 19.4	77.4 ± 23.3	78.0 ± 18.6	45.2 ± 22.3#
**Fresh controls (n = 40)**	210.6 ± 32.4	90.7 ± 7.8	86.3 ± 7.3	90.9 ± 7.1	88.8 ± 6.6	88.1 ± 6.8	58.5 ± 20.4#

The Table reflects aggregation responses of patients diagnosed with GT, BSS, SSD, parallel controls (drawn simultaneously to patients), and controls drawn at the time of analysis. All values are mean ± standard deviation. *Always <20 × 10^9^ platelet L^−1^; #Aggregation responses to PMA were assessed in controls only when the paired patient’s aggregation profile was consistent with GT. ‡Blood samples from nine patients diagnosed with secretion and signal transduction platelet defects were drawn at our institution, and in those cases blood specimens were only collected from fresh (not parallel) controls.

*Abbreviations*: *ADP* Adenosine diphosphate, *BSS* Bernard Soulier Syndrome, *GT* Glanzmann Thrombasthenia, *TRAP* thrombin receptor activating peptide, *PMA* phorbol myristate acetate, *SSD* platelet secretion and signal transduction defects.

Patients presented with a variable bleeding diathesis as measured by the BS (mean BS of 10.5, Table 1). Considering grades 2 and 3 as clinical significant bleeding, most of the patients had gross blood loss due to epistaxis (54%), cutaneous bleeding (58%) and menorrhagia (100% of women), while other common symptoms (oral cavity, gastrointestinal, following dental extraction or mild injuries, postpartum, or hematomas) were in general milder.

In order to determine the causative mutations, *ITGB3* and *ITG2B* genes were analyzed, allowing us to identify mutations in all patients but one (Table 3). Overall, 11 mutations were found in *ITGA2B* and 5 in *ITGB3*. Of the 16 alterations, 8 were novel mutations, including 2 nonsense mutations (leading to premature stop codons for amino acids 51 and 507 in α_IIb_), 3 frameshift changes in *ITGA2B* (c.2637delC, c.2965delG, and c.2473_2481delinsTCACCTGGTC) and one in *ITGB3* (c.774-775delTG), one mutation affecting a splice site c.1599delAT in *ITGA2B*, and one missense p.Tyr190Cys in *ITGB3*; the other 8 mutations were already described in the literature [26-32]. As GT is an autosomal recessive disease, patients are mostly compound heterozygotes for *ITGA2B* or *ITGB3* mutations in the absence of consanguinity. Homozygous mutations were found in three patients (GT-3, GT-9, and GT-13) from non-consanguineous families -to the best of our knowledge, and in all patients with consanguineous family history. Despite complete sequencing, no potential pathological mutations were located in either gene of patient GT-12, who exhibited a family history of consanguineous union, and presented no detectable levels of *ITGB3* mRNA by qRT-PCR (data not shown).

Segregation analysis confirmed the presence of heterozygous mutations in all available carriers (parents) except patient GT-6. Interestingly, patient GT-6 exhibited a triple heterozygosity in the integrin α_IIb_ subunit, as a result of a nonsense mutation (p.Glu507Stop) of paternal origin, and two *de novo* missense changes, p.Val951Met, and p.Ala958Thr, that had been previously described in another GT patient [31]. Noteworthy, the already reported missense mutations- found together with a splice site mutation in the α_IIb_ gene inherited from the mother- were paternal in origin and cosegregated in the same allele across three generations [31]. In our patient, however, throughout investigation of other family members (father, mother, brother) did not detect the two missense mutations in any of the individuals, and DNA profiles generated from blood from the patient and parents revealed double paternal/maternal contribution at different loci in the propositus, confirming the maternal and parental relationship.

To define the structural impact of the new missense mutation p.Tyr190Cys, identified in two of our patients (patients GT-4 and GT-11), the role of the amino acid substitutions in β_3_ was analyzed *in silico*. The three dimensional diagram of the Cys190 suggested a disruption of the hydrogen bond created in between Tyr190 and Tyr115. This change would also generate new hydrogen bond with Gly189, predicting a destabilizing effect on the fold (data not shown).

### Bernard Soulier syndrome

This syndrome was diagnosed in eight individuals (2 men and 6 women with a median age of 40 years) (Tables 1 and 5). In these patients, platelet count was consistently less than 20 × 10^9^ L^−1^ by commercial cell counters, with persistent findings of giant platelets in the peripheral blood smear. Patients had slightly diminished aggregation responses to TRAP compared to their parallel controls, but RIPA was absent or severely defective in all patients (Table 4). Flow cytometry revealed that the GPIbα and GPIX expression was severely reduced (<10% compared to controls). MFI of other platelet GPs were normal or increased due to the increased size of the cells. Further studies using Western-blot analysis revealed an absent or severely reduced GPIbα band in all patients (data not shown).

**Table 5:** **Molecular diagnosis of Bernard Soulier syndrome patients**

**Patients with molecular diagnosis**	**Gene**	**Inheritance**	**Mutation**	**Reference**
**BSS-1**	*GP9*	Homozygous	p.Trp71Arg	new
**BSS-2**	*GP1BA*	Homozygous	p.Cys209Ser	[34]
**BSS-3**	*GP1BA*	Possibly Heterozygous	p.Gln90_Leu92del	new
			-	-
**BSS-4**	*GP9*	Homozygous	p.Asn45Ser	[33,36]
**BSS-5**	*GP9*	Homozygous	p.Phe55Ser	[35]
**BSS-6**	*GP9*	Homozygous	p.Phe55Ser	[35]
**BSS-7**	*GP9*	Homozygous	p.Phe55Ser	[35]
**BSS-8**	*GP1BB*	Homozygous	p.Pro90Ser	new

*Abbreviations*: *BS* bleeding score, *BSS* Bernard Soulier Syndrome.

More than half of the patients (BSS-2, BSS-5, BSS-6, BSS-7 and BSS-8) had been splenectomized without having any benefit, as they had been previously misdiagnosed with primary immune thrombocytopenia (ITP). The severity grade, measured by BS for epistaxis and menorrhagia was 2 & 3 in 100% of patients. Other less prevalent locations of moderate-severe blood loss were cutaneous bleeding (50% of patients), and following procedures of dental extraction, surgery, or delivery (57%, 71%, and 75% of patients, respectively). Mild bleeding symptoms were reported at other sites (bleeding from minor wounds, oral cavity, or gastrointestinal tract). The overall BS in BSS patients was 11.8 (Table 1).

Disease-causing homozygous mutations were found in 7 out of 8 patients -five lacking a history of known family consanguinity-, while in the remaining patient a mutation was identified in only one allele of *GP1BA* (Table 5). Six mutations were detected (3 in *GP9*, 2 in *GP1BA*, and 1 in *GP1BB*); half of them had been already described in the literature [33-36] and the other 3 were new (one in *GP1BA*, one in *GP1BB*, and one in *GP9*). Of these novel mutations, 2 of them were missense, Trp71Arg in exon 3 of *GP9*, and Pro64Ser in exon 2 of *GP1BB*. Of note, the p.Gln90_Leu92del mutation, which leads to the loss of 3 amino acids in one of the leucine rich-repeat sequences of GPIbα, was found in heterozygosis in one patient. We did not consider this patient as having the milder monoallelic autosomal dominant form of BSS [37-40], due to the absent GPIbα and GPIX expression by flow cytometry, and the low platelet count (<20 × 10^9^ L^−1^). Additionally, this variant was also identified in the patient’s mother, with no BSS phenotype at all. Hence, there might be other unknown genetic defects or different molecular mechanisms, such as transcription factors or post translational defects [41] that could lead to BSS phenotype in this patient. It is worth mentioning that the variant p.Phe55Ser, previously described in a patient from the Netherlands [35] was identified in 3 unrelated patients from the central region of Portugal, suggesting that this mutation could have an ancient origin in that geographical area.

To predict the effects of the 2 novel missense mutations, we performed *in silico* analysis of the new variants. The analysis of the p.Trp71Arg variant in GPIX reflected a change in the isoelectric point that could affect the interaction with GPIbβ, potentially influencing the correct folding and expression of the receptor on the platelet membrane. As for the p.Pro64Ser in GPIbβ, *in silico* studies did not show any relevant alteration of the protein structure but mutation analysis using different bioinformatics tools (Poly-Phen2, Mutation Taster) characterized this change as a damaging variant for the protein and a disease causing mutation (data not shown).

### Other IPDs

The molecular characterization of patients with the clinical suspicion of other IPDs resulted in the identification of 8 molecular alterations, 3 recently reported by us [42,43]. We did not confirm any of the 2 PvWD suspicions. Two homozygous mutations in *LYST* corroborated the diagnosis of CHS in 2 patients: p.Cys258Arg and p.Gly3725Arg [42], while HPS was confirmed in one patient (p.Glu204Stop) [43]. Three monoallelic mutations, already described in the literature [44,45], were detected in MYH9 patients: 2 patients had been referred to our center with the clinical suspicion of MYH9 (p.Arg1165Cys, p.Glu1841Lys), while the remaining individual had been clinically misdiagnosed as having BSS. In this patient with macrothrombocytopenia and no evident neutrophil inclusions on conventional May-Grünwald-Giemsa-stained blood, whole exome sequencing detected the p.Asp1424Glu variant in *MYH9* [45]. Molecular diagnosis of CAMT was attained in one patient by the identification of the homozygous p.Arg102Cys [23] mutation in *MPL*. THC2 was diagnosed in an additional patient with a clear family history of autosomal dominant thrombocytopenia with normal MPV, as the monoallelic c.-128G > A variant [24] in the 5′UTR region of the *ANKRD26* gene was detected*.* (Table 6). Patients presenting with non-syndromic normal or mildly reduced platelet counts, decreased aggregation responses to collagen, and/or ADP, and impaired externalization of platelet activation markers in response to TRAP were lumped in the remarkably heterogeneous group of platelet secretion and signal transduction defects, and no further analysis were performed.

**Table 6:** **Molecular diagnosis of other inherited platelet disorders**

**Patients with molecular diagnosis**	**Gene**	**Inheritance**	**Mutation**	**Reference**
**Chediak-Higashi Syndrome (CHS)**				
**CHS-1**	*LYST/CHS1*	Homozygous	p.Gly3725Arg	[42]
**CHS-2**	*LYST/CHS1*	Homozygous	p.Cys258Arg	[42]
**Hemansky-Pudlak Syndrome (HPS)**				
**HPS-1**	*HPS1*	Homozygous	p.Glu204Stop	[43]
**MYH9 related disorders (MYH9-RD)**				
**MYH9-RD-1**	*MYH9*	Heterozygous	p.Arg1165Cys	[44]
**MYH9-RD-2**	*MYH9*	Heterozygous	p.Glu1841Lys	[44]
**MYH9-RD-3**	*MYH9*	Heterozygous	p.Asp1424Glu	[45]
**Congenital Amegakaryocytic Thrombocytopenia (CAMT)**				
**CAMT-1**	*MPL*	Homozygous	p.Arg102Cys	[23]
**Thrombocytopenia 2 (THC2)**				
**THC2-1**	*ANKRD26*	Heterozygous	c.-128G > A	[24]

## Discussion

In this study, we evaluated the largest cohort of BSS and GT patients in the Iberian Peninsula, following the aim of the project “Functional and Molecular Characterization of Patients with Inherited Platelet Disorders”. By reviewing the medical records, BS questionnaire, performing functional studies, and when possible, identifying the underlying genetic abnormality, amongst the 70 patients referred to us for a diagnostic confirmation of IPD, we concluded that 32.9% had been previously misdiagnosed, while 40% achieved a diagnosis at a molecular level (Table 1).

IPDs are one of the most complex, poorly standardized and time consuming disorders to diagnose. A recent worldwide survey [46] confirms results from previous ones [47,48] showing large variations between laboratories in platelet function testing practice, and clearly demonstrates that the required diagnostic technologies are not always readily available to many facilities, and referral to specialized centers may be necessary for definitive diagnosis [49]. Even though sending samples to a distant laboratory might pose the problem of missing mild platelet defects, due to the time lag between blood extractions and testing, differences in platelet functional responses to strong agonists between patients and paired controls allowed us not only to functionally identify GT and BSS patients, but also to characterize individuals within the heterogeneous category of defects in platelet secretion and signal transduction.

In that sense, we restricted the study to patients with significant bleeding history. The selection of candidate patients justifies the differences in the prevalence of confirmed IPD between our study and the previous recent worldwide survey [46], with a higher percentage of patients with GT (28.8% vs. 9.8%, respectively), with BSS (17.7% vs. 4.2%, respectively), and the percentage of patients that achieve a diagnosis at a molecular level (40% vs. 8.7, respectively). A reflection of the need to improve the recognition of IPDs and the absence of referral centers is that 93% of the patients investigated at our institution were adults, and although IPD was suspected during childhood (median age of 12 years), laboratory confirmation of the disorder was not performed until a median of 23 years later. Additionally, even in GT and BSS, defects in which the diagnosis is rather straightforward, even for non-specialized laboratories, the percentage of patients that had been misdiagnosed was high. The long delay in reaching a definite diagnosis was not without consequences, since 8 subjects underwent undue splenectomy before our observation (2 with MYH9-related disease, 5 with BSS, 1 with CAMT).

The analysis of the 13 GT patients showed a consistent and homogeneous functional profile and bleeding complications leading to significant morbidity. Most patients had a type I phenotype, in accordance with previous reports [50,51]. The molecular characterization of patients revealed 16 mutations (half of them not reported previously) along both genes encoding for the α_IIb_β_3_ receptor: 11 mutations were located in the *ITGA2B* gene within 8 patients (3 of them were compound heterozygous) and 5 in the *ITGB3* in 4 more patients. According to a regularly updated database (http://sinaicentral.mssm.edu/intranet/research/glanzmann), and to what has been previously stated [15], we observed more mutations affecting *ITGA2B* possibly because, although a smaller gene than *ITGB3*, it has 30 exons (and double number of splice sites) compared to 15 in *ITGB3*. In patient GT-12, who has unequivocally been diagnosed by functional studies as GT, no mutations were detected. It is known that no single screening technique warrants detection of all causative mutations; previous reports have identified mutations in 80% of GT patients, by using different techniques, such as denaturing gradient gel electrophoresis [52], or by conformation sensitive gel electrophoresis [51]. This suggests that defects in regulatory elements that adversely affect the transcription of the *ITGA2B* and *ITGB3* genes or abnormalities in mechanisms that are responsible for post-translational modifications and trafficking of integrin subunits may also potentially account for some cases of GT.

The detected mutations were analyzed for penetrance into the individual family members; heterozygous mutations were seen in all of the available samples from the parents of GT, except in one case. Of note, patient GT-6 exhibited two *de novo* missense variants in the α_IIb_ subunit, together with a nonsense mutation of parental origin. Such missense mutations have been previously described in a patient also with triple heterozygosity in the α_IIb_ gene [31], indicating that both missense mutations, 8 aminoacids from each other, occur non-randomly but that these variants are tightly linked. At this moment, the causative molecule or defect in the repair mechanism responsible for these *de novo* mutations are not known. While one of the missense variants (p.Ala958Thr) was considered to be a polymorphism in the previous report [31], using exome databases (Exome Variant Server, NHLBI GO Exome Sequencing Project (ESP), Seattle, WA (URL: http://evs.gs.washington.edu/EVS/)), this change was identified as a mutation (allele frequency < 0.01). Further studies are needed to clarify the linkage disequilibrium between both mutations in exon 29 of *ITGA2B*.

BSS was diagnosed in 8 individuals presenting with macrothrombocytopenia, decreased/absent levels of GPIbα and GPIX, and a bleeding tendency with variable severity among individuals. Similar to GT, all menstruating women had severe menorrhagia (grades 2 & 3), a finding that might justify the predominance of females (n = 15) over males (n = 6) in these two disorders (47 vs 23 considering the whole cohort), and suggesting that men with GT, BSS, or IPDs in general, are being underdiagnosed. All cases but one (patient BSS-3) were homozygous for the same mutated allele inherited from parents, inferring that these patients shared a common ancestor (5 out of 8 did not refer a history of known family consanguinity). This is of particular relevance in the case of the variant p.Phe55Ser in GPIX, identified in 3 apparently unrelated patients from central Portugal.

It has been previously shown that missense mutations hit GPIbβ and GPIX more frequently than GPIbα, suggesting that folding and stability of these glycoproteins are more sensitive to aminoacid substitution than GPIbα [53]. Accordingly, of the 3 new mutations identified in our study, the 2 novel missense variants, p.Trp71Arg, and p.Pro64Ser affected GPIX, and GPIbβ, respectively. Structural modeling of these variants predicted deleterious effects.

Until 2011, original studies reported one or two patients with BSS, with the largest case series including 13 cases [41]. More recently, the BSS Consortium, constituted of research groups from 15 countries worldwide, has helped establish the most comprehensive database of BSS, and data from the 112 different variants characterized so far have been deposited in the Leiden Open Variation Database (LOVD) at http://www.lovd.nl/GP1BA, http://www.lovd.nl/GP1BB, and http://www.lovd.nl/GP9 for the *GP1BA*, *GP1BB*, and *GP9* genes, respectively [53]. Such database would be enriched, with the inclusion of the 3 novel mutations identified here.

As for other IPDs, it is known that the molecular diagnosis of HPS may not be that simple. Mutations in 9 genes (*HPS1, AP3B1, HPS3, HPS4, HPS5, HPS6, DTNBP1, BLOC1S3*, and *BLOC1S6*) can cause HPS in humans. By adopting a classical approach to reach a molecular diagnosis in the 2 patients with clinical suspicion of HPS, we selected *HPS1* as the first candidate gene to be responsible for the disease, since it is the one most frequently affected [54]. We identified a homozygous p.Glu204Stop mutation in exon 7 of this gene in one of the patients [43]. In the second patient, further sequencing of *HPS4* revealed no variations; we did not sequence the rest of less frequently affected genes. Two additional patients presented with syndromic forms, suggestive of CHS, and a definitive diagnosis was made based on molecular findings [42]. One individual who had been previously misdiagnosed as having ITP and treated with steroids and splenectomized, exhibited a bone marrow aspirate and biopsy showing decreased cellularity and megakaryocyte counts, with 24% of the metaphases revealing monosomy 7. With the clinical suspicion of CAMT, sequencing of the *MPL* gene detected a homozygous point mutation, p.Arg102Cys previously described [23]; with a diagnosis of CAMT the patient underwent a matched unrelated bone marrow transplant. A woman with non-syndromic autosomal-dominant thrombocytopenia was categorized as having a THC2, due to the presence of a heterozygous c.-128 G > A ANKRD26 mutation. One patient, who was initially thought by the referring physician to be affected by BSS, was not confirmed by functional studies as having such disease (normal expression of GPIbα and GPIX, as well as platelet agglutination induced by ristocetin). In this patient, in whom MYH9-related disease was not suspected due to (1) no evident neutrophil inclusions on conventional May-Grünwald-Giemsa-stained blood, (2) no family history of macrothrombocytopenia, and (3) no extra-hematological manifestations, the use of exome sequencing, led us to identify a monoallelic mutation in the *MYH9* gene (p.Asp1424Glu). This observation supports the possibility that exome sequencing could be used as a diagnostic tool in the future, to help identify genetic defects in patients who do not fall into known categories of IPDs, and to have a deeper knowledge of the molecular and genetic mechanisms underlying functional defects of patients currently categorized as having signaling and/or secretion disorders.

## Conclusion

This project highlights the urgent need to significantly improve the diagnosis of IPDs at the level of the platelet pathway and by molecular analysis, considering the high percentage of patients that are still being misdiagnosed, and those in whom, the molecular mechanisms causing these defects are still unknown. The requirement of highly specialized tests represents a particular problem in certain institutions or areas, and collaborative international efforts should facilitate advances in patient diagnosis and care. Consensus standardized criteria for the diagnosis of these disorders are expected to overcome the present heterogeneity between facilities, and the creation of reference centers should be considered to help health care providers in the diagnosis of these disorders.
